# A review of brain regions and associated post-concussion symptoms

**DOI:** 10.3389/fneur.2023.1136367

**Published:** 2023-08-03

**Authors:** Ethan Danielli, Nicholas Simard, Carol A. DeMatteo, Dinesh Kumbhare, Stephan Ulmer, Michael D. Noseworthy

**Affiliations:** ^1^School of Biomedical Engineering, McMaster University, Hamilton, ON, Canada; ^2^Imaging Research Centre, St. Joseph's Healthcare Hamilton, Hamilton, ON, Canada; ^3^KITE Research Institute, Toronto Rehabilitation Institute, University Health Network, Toronto, ON, Canada; ^4^Department of Electrical and Computer Engineering, McMaster University, Hamilton, ON, Canada; ^5^ARiEAL Research Centre, McMaster University, Hamilton, ON, Canada; ^6^Department of Rehabilitation Sciences, McMaster University, Hamilton, ON, Canada; ^7^Division of Physical Medicine and Rehabilitation, Department of Medicine, University of Toronto, Toronto, ON, Canada; ^8^Neurorad.ch, Zurich, Switzerland; ^9^Department of Radiology and Neuroradiology, University Hospital of Schleswig-Holstein, Kiel, Germany; ^10^Department of Radiology, McMaster University, Hamilton, ON, Canada

**Keywords:** post-concussion symptoms, brain anatomy, concussion, gray matter, white matter, cerebellum, superior longitudinal fasciculus, insula

## Abstract

The human brain is an exceptionally complex organ that is comprised of billions of neurons. Therefore, when a traumatic event such as a concussion occurs, somatic, cognitive, behavioral, and sleep impairments are the common outcome. Each concussion is unique in the sense that the magnitude of biomechanical forces and the direction, rotation, and source of those forces are different for each concussive event. This helps to explain the unpredictable nature of post-concussion symptoms that can arise and resolve. The purpose of this narrative review is to connect the anatomical location, healthy function, and associated post-concussion symptoms of some major cerebral gray and white matter brain regions and the cerebellum. As a non-exhaustive description of post-concussion symptoms nor comprehensive inclusion of all brain regions, we have aimed to amalgamate the research performed for specific brain regions into a single article to clarify and enhance clinical and research concussion assessment. The current status of concussion diagnosis is highly subjective and primarily based on self-report of symptoms, so this review may be able to provide a connection between brain anatomy and the clinical presentation of concussions to enhance medical imaging assessments. By explaining anatomical relevance in terms of clinical concussion symptom presentation, an increased understanding of concussions may also be achieved to improve concussion recognition and diagnosis.

## 1. Introduction

The field of concussion awareness, prevention, and mitigation is constantly growing. As a result, the medical knowledge of anatomical and physiological changes post-concussion is still evolving. To improve concussion diagnosis and personalized treatment, it is important to first understand brain structures and their respective functions. This review briefly defines concussion characteristics, but the main focus is on functional brain anatomy and the relationship of specific damaged brain regions to resulting post-concussion symptoms. The human brain is an exceptionally complex organ that comprises billions of neurons (1). Our brain consists of a large cerebrum, with left and right hemispheres made up of four lobes (frontal, temporal, parietal, and occipital), central sub-cortical structures (2), and the cerebellum (Latin for “little brain”) (3). The cortical surface, or gray matter, contains the neuronal cell bodies, dendrites, glial cells, axons, and synapses that produce neuronal signals and are found in the cortical, sub-cortical, and cerebellar areas as the gray layer (2). Conversely, white matter contains myelinated and unmyelinated neuronal axons, which are the physical connection between neuronal cell bodies that transmit the neuronal signals efficiently between gray matter regions (2). The cerebellum is an immensely folded brain region, segmented from the cerebrum, that is involved in all aspects of human function and cognition (3).

Concussions are complex injuries that can have various acute and chronic complications (4). A concussion is caused by a blow to the head, neck, or body that results in the brain becoming injured by resultant propagating forces (rapid de- or acceleration) and does not have to involve a loss of consciousness (4). Most adults who sustain a concussion recover within 10–14 days (~90%); however, many people have symptoms persisting longer than a month (4–6). Concussions are mainly caused by motor vehicle accidents, falls, assaults, and sports, with domestic events and non-professional sports being the leaders of this injury (5, 7). The forces applied to the brain during concussive events can produce serious shearing and tearing of tissues that trigger a cascade of neurometabolic changes (8). These structural, functional, and physiological brain alterations manifest uniquely in each individual, where these pathophysiological alterations are often undetectable on conventional clinical neuroimaging exams because these changes are on the molecular or microvascular scale. A head injury that results in structural injuries such as brain bleeding and swelling or a skull fracture would classify as a more severe traumatic brain injury and not a concussion. Thus, concussion-related brain damage or injury is most often present as shearing or tearing of white matter tracts (9) that result in the ionic dysregulation of sodium-potassium, impaired neurotransmission along axons (10), or more functional implications such as abnormal cerebral blood flow (11) and decreased blood-oxygen level-dependent (BOLD) signal, measured using functional magnetic resonance imaging (fMRI) scans (12), of focal brain regions (13) and across functional networks (14). With that information, this review will remain unspecific to the root injury pathophysiology (e.g., ionic dysregulation, decreased cerebral blood flow, or neurovascular uncoupling) when describing concussion symptoms to concussion-related brain damage. Furthermore, this review will focus on specific and focal brain regions as functional brain networks deserve a review of their own.

## 2. Regional brain anatomy and associated post-concussion symptoms

**Figure 1:**
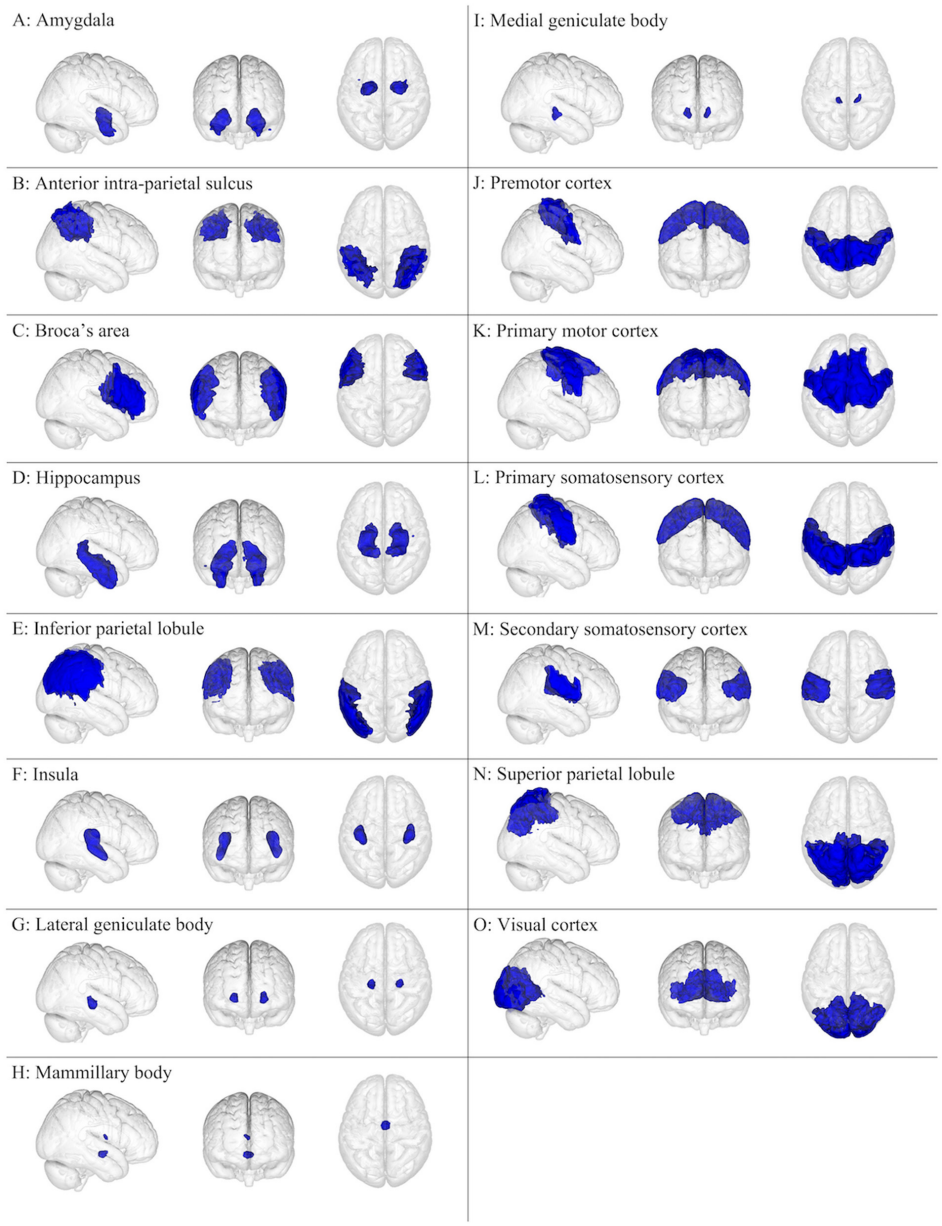
Gray matter brain regions (colored blue) relevant to concussion-related damage that is organized as **(A–O)**: **(A)** amygdala, **(B)** anterior intra-parietal sulcus, **(C)** Broca's area, **(D)** hippocampus, **(E)** inferior parietal lobule, **(F)** insula, **(G)** lateral geniculate body, **(H)** mammillary body, **(I)** medial geniculate body, **(J)** premotor cortex, **(K)** primary motor cortex, **(L)** primary somatosensory cortex, **(M)** secondary somatosensory cortex, **(N)** superior parietal lobule, and **(O)** visual cortex. These brain regions are overlayed onto the MNI152 1 mm standard space T1-weighted brain from the (left to right) right sagittal, anterior frontal, and superior axial perspectives. These brain regions were from the Juelich Histological atlas (15–17).

**Figure 2:**
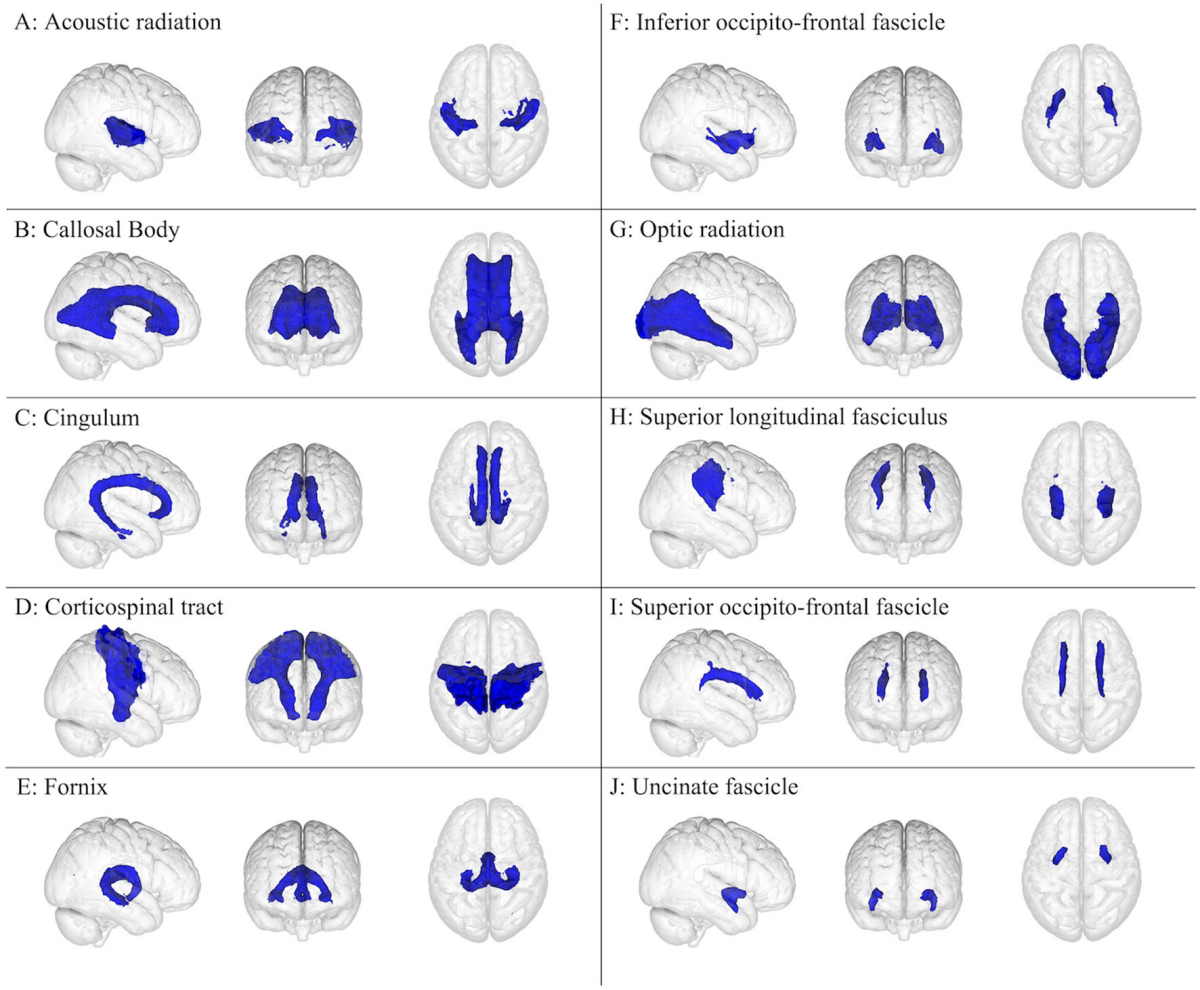
White matter brain regions (colored blue) relevant to concussion-related damage that is organized as **(A–J)**: **(A)** acoustic radiation, **(B)** callosal body, **(C)** cingulum, **(D)** corticospinal tract, **(E)** fornix, **(F)** inferior occipito-frontal fascicle, **(G)** optic radiation, **(H)** superior longitudinal fasciculus, **(I)** superior occipito-frontal fascicle, and **(J)** uncinate fascicle. These brain regions are overlayed onto the MNI152 1 mm standard space T1-weighted brain from the (left to right) right sagittal, anterior frontal, and superior axial perspectives. These brain regions were from the Juelich Histological atlas (15–17) and the JHU DTI-based white matter atlases (18–20).

**Figure 3:**
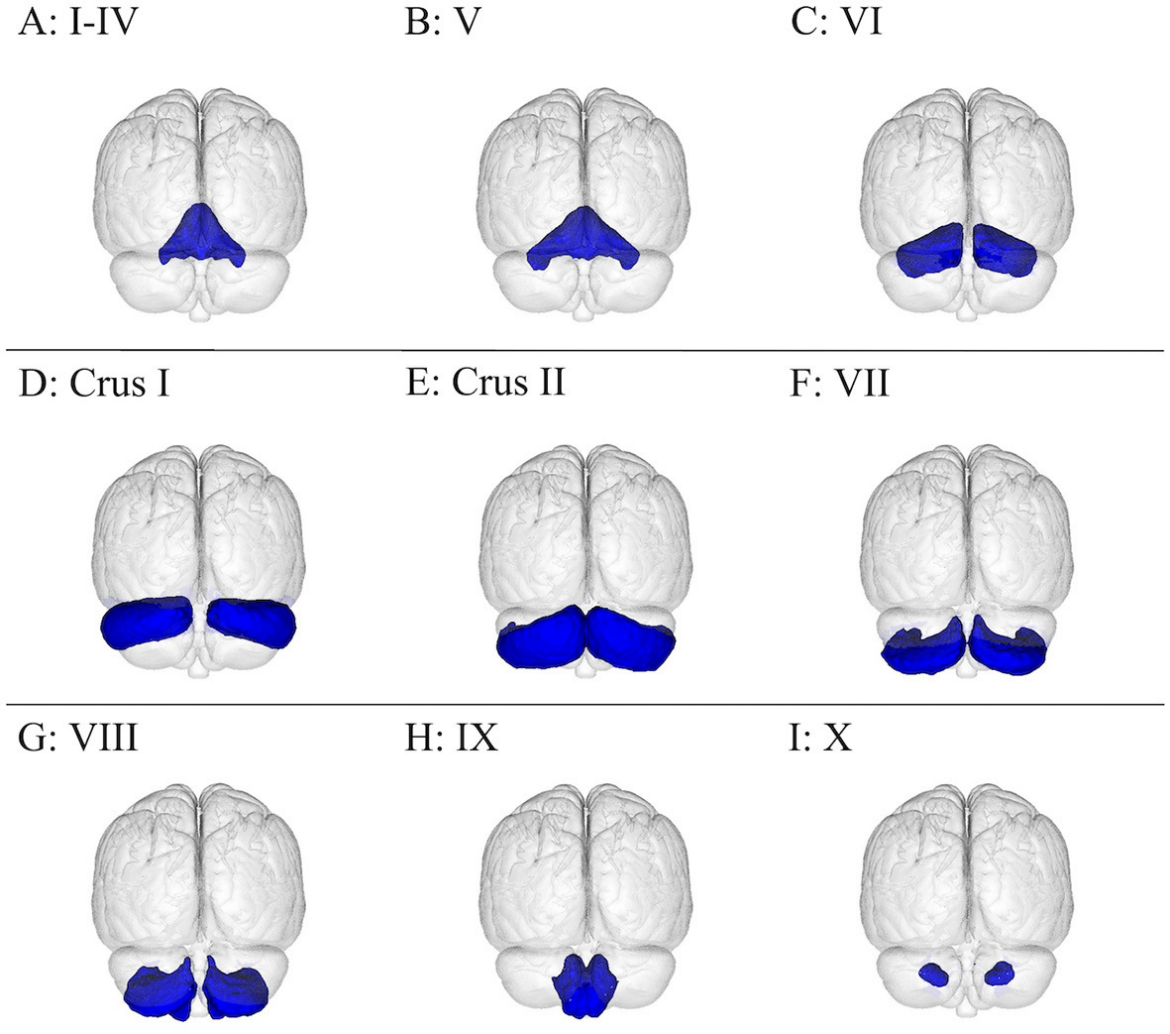
Visualization of the cerebellum subdivisions overlayed onto the MNI152 1 mm standard space T1-weighted brain from the posterior, frontal plane perspective. There are 12 cerebellar regions in this figure, with lobules I–IV amalgamated, organized as follows: **(A)** I–IV, **(B)** V, **(C)** VI, **(D)** crus I, **(E)** crus II, **(F)** VII, **(G)** VIII, **(H)** IX, and **(I)** X. These brain regions were from the Probabilistic (FNIRT) cerebellar atlas (26).

The brain has been the subject of extensive research, typically conducted as anatomical dissection, histology, and medical imaging, but also as cell/tissue culture and biochemical/genetic assays, which have allowed for an ever-improving understanding of normal and pathological brain function. This review focuses on 15 cerebral gray matter (Figure 1) and 10 cerebral white matter brain regions (Figure 2) that are physically large and have been shown in the literature to have important and specific functional relevance to concussions (21–25). The cerebellum and its subdivisions (Figure 3) were also examined to discuss its involvement in post-concussion symptoms, emphasize the influence it has over neurocognitive function, and encourage increased clinical and research attention to this important but often overlooked part of the human brain. This review is a non-exhaustive compilation of brain regions and concussion symptoms and is intended to be a reference point for researchers and clinicians. Neuroplasticity can alter regional brain function in specific individuals, especially post-injury, but the inclusion of all possible neuroplastic possibilities falls outside the scope of this review.

Brain regions will be discussed in terms of their location within the human brain, their healthy functional involvement, and common alterations found post-concussion expressed as somatic, cognitive, emotional, or sleep-related symptoms (4, 27, 28) (Table 1). Somatic post-concussion symptoms can include headaches, nausea, vomiting, balance problems, visual problems, dizziness, light-headedness, fatigue, sensitivity to light, sensitivity to noise, numbness, tingling, bodily pain, and motor control problems. Cognitive post-concussion symptoms can include feeling “slow”, feeling “foggy”, difficulty concentrating, difficulty remembering, confusion, repetitive speech, and language problems. Emotional post-concussion symptoms can include irritability, sadness, feeling hopeless, nervousness, anxiousness, and feeling more emotional. Finally, sleep-related post-concussion symptoms can include trouble falling asleep, sleeping more or less than usual, and drowsiness. The variety of post-concussion symptoms indicates how a range of brain regions could be implicated during a single concussive injury, and why damage to specific brain regions may explain patient-specific symptoms.

**Table 1:** A summary table for the four primary categories of post-concussion symptoms—somatic, cognitive, emotional, and sleep—along with a list of the corresponding post-concussion symptoms within each category.

**Symptom category**	**Post-concussion symptoms**
Somatic	• Headaches • Nausea • Vomiting • Balance problems • Visual problems • Dizziness • Light-headedness • Fatigue • Sensitivity to light • Sensitivity to noise • Numbness • Tingling • Bodily pain • Motor control problems
Cognitive	• Feeling “slow” • Feeling “foggy” • Difficulty concentrating • Difficulty remembering • Confusion • Repetitive speech • Language problems
Emotional	• Irritability • Sadness • Feeling hopeless • Nervousness • Anxiousness • Feeling more emotional
Sleep	• Trouble falling asleep • Sleeping more or less than usual • Drowsiness

### 2.1. Gray matter brain regions

#### 2.1.1. Amygdala

The amygdala is a symmetric deep brain structure that comprises a group of neurons located antero-medial to the hippocampus and sub-cortical to the temporal lobe (Figure 1A). It is almond-shaped and subdivided into the centro-medial, latero-basal, and superficial groups (29). The main role of the amygdala involves emotional and cognitive processing linked to the limbic system (30–32). Emotional responses related to pain, fear, incoming threats, reward-related activities, empathy, personal importance/significance, and facial expressions are all governed by the amygdala (33–36). Moreover, the amygdala has been noted to play roles in social attention, social responses, salience tagging, interpreting visual signals, tactile learning, explicit memory, and implicit learning (29, 37).

Damage to the amygdala can lead to deficits in emotional processing, emotional learning, and memory, which can be further manifested in autism spectrum disorder, psychopathy, and loss of the “cognitive control” system in adolescents (36, 38–42). Furthermore, sensitivity to fearful facial expressions, fear conditioning to social responses, alterations in vigilance, reduced self-motivation, and deficits in socio-emotional function can be caused by a damaged amygdala (29, 36, 43). This was corroborated in adolescents with persistent concussion symptoms who had elevated incidence of emotional/behavioral symptoms (e.g., depression, anxiety, and anhedonia) with decreased amygdala activity in response to an emotional face-processing task (44). With respect to sleep, a study using [18F]-fluorodeoxyglucose positron emission tomography (FDG PET) to measure the effect of combat-caused mild traumatic brain injuries on the relative cerebral metabolic rate of glucose (rCMRglc) found that head trauma was associated with lower rCMRglc while awake and during rapid-eye movement (REM) sleep in the amygdala, hippocampus, parahippocampal gyrus, thalamus, insula, uncus, culmen, visual association cortices, and midline medial frontal cortices (45). Based on the previously mentioned research on healthy and injured amygdala function, the common post-concussion symptoms related to the amygdala could result in somatic [e.g., headaches (46)], cognitive [e.g., feeling “slow” or “foggy”, difficulty concentrating, or difficulty remembering (29, 37)], emotional [e.g., irritability, sadness, nervousness, more emotional (33, 44)], or sleep symptoms [e.g., trouble falling to sleep, loss of sleep (45)] (Table 2).

**Table 2:** A summary of 15 gray matter brain regions and their associated functions and concussion-related symptoms.

**Brain region**	**Associated functions**	**Concussion-related symptoms**
Amygdala	• Emotional processing and learning • Memory • Fearfulness sensitivity • Self-motivation • Socio-emotional function	• Headache • Feeling “slow” or “foggy” • Difficulty concentrating • Difficulty remembering • Irritability • Sadness • Nervousness • More emotional • Trouble falling asleep • Loss of sleep
Anterior intra-parietal sulcus	• Visuomotor functions • Finger manipulation • Tactility • Eye movements • Vestibular and egocentric attention • Auditory coordinate location • Hierarchical structure processing • Memorizing geometry • Affected by light levels	• Light sensitivity • Noise sensitivity • Motor control problems • Visual problems • Feeling “slow”
Broca's area	• Language processing • Motor control with speech	• Feeling “slow” or “foggy” • Difficulty concentrating • Difficulty remembering • Speech and language problems
Hippocampus	• Every aspect of memory • Learning new tasks • Understanding verbal and spatial cues • Motivation • Executive function	• Feeling “foggy” • Difficulty remembering • Feeling more emotional • Sadness • Nervousness
Inferior parietal lobule	• Visuospatial navigation • Sound perception • Auditory memory • Saccadic eye movements • Egocentric decision-making • Emotional empathy • Speech and language processing • Reading and writing	• Headache • Balance problems • Dizziness • Light Sensitivity • Noise Sensitivity • Visual problems • Motor control problems • Feeling “slow” or “foggy” • Difficulty concentrating • Difficulty remembering
Insula	• Somatic, motor, and emotion • Tactility • Auditory • Taste • Pain • Speech production • Gastric motility • Patterned motor movements • Cardiovascular function • Decision-making tasks	• Headache • Nausea • Vomiting • Sensitivity to noise • Bodily pain • Feeling “slow” or “foggy” • Language problems • Irritability • Feeling more emotional
Lateral geniculate body	• Relay of visual input	• Visual problems • Sensitivity to light • Sensitivity to noise
Mammillary body	• Memory • Olfactory	• Headache • Loss of smell
	• Inability to understand smells • Spatial abilities	• Difficulty remembering
Medial geniculate body	• Auditory processing • Speech comprehension	• Headache • Sensitivity to noise • Nervousness
Premotor cortex	• Generate and plan motor movements • Proprioception and spatial awareness • Fine and gross motor coordination	• Headache • Nausea • Balance problems • Dizziness • Fatigue • Motor control problems • Feeling “slow” • Difficulty remembering • Language problems
Primary motor cortex	• Motor control • Movement execution • Sensory feedback	• Headache • Nausea • Vomiting • Balance problems • Fatigue • Motor control problems • Feeling “slow”
Primary somatosensory cortex	• Interpretation of all sensory information • Distinct localization of where sensory input originated	• Headache • Nausea • Vomiting • Balance problems • Sensitivity to light • Sensitivity to noise • Bodily pain • Numbness • Visual problems
Secondary somatosensory cortex	• Secondary processing and interpretation of sensory information	• Headache • Nausea • Vomiting • Balance problems • Dizziness • Sensitivity to light • Sensitivity to noise • Numbness • Visual problems • Bodily pain
Superior parietal lobule	• Somatosensory and visual interpretation for specific motor movements • Egocentric tasks • Emotion-relevant behavior • Auditory association	• Headache • Balance problems • Dizziness • Sensitivity to light • Motor control problems • Visual problems • Feeling “slow” • Difficulty remembering • Confusion
Visual cortex	• Processing of all visual information • Colors, shapes, motion, and light	• Headache • Nausea • Vomiting • Balance problems • Dizziness • Sensitivity to light • Visual problems

#### 2.1.2. Anterior intra-parietal sulcus lobule (anterior wall of the intraparietal sulcus)

The anterior intra-parietal sulcus lobule occupies the antero-lateral bank of the deep intraparietal sulcus that spans the surface of the parietal lobe (Figure 1B) (29). The anterior intra-parietal lobule can be further subdivided into three zones (hlP1, hlP2, and hlP3) based on cytoarchitecture (47, 48). Regions hlP1 and hlP2 are situated in the lateral wall of the anterior intra-parietal sulcus, while the hlP3 region lies more medial and has a distinctly different laminar pattern from the rest of the other superior parietal lobe gray matter, which ends posteriorly at the base of the intra-parietal sulcus (48). The anterior intra-parietal sulcus lobule communicates with the cingulum (33), superior longitudinal fasciculus (49), sensory and motor cortices (50, 51), insula (52), the temporal (50) and occipital lobes (53), and neighboring parietal structures such as the inferior and superior parietal lobules (48, 52).

The anterior intra-parietal sulcus lobule mainly contributes to visuomotor functions including finger manipulation (49, 51), tactility (53), eye movements (51, 54), vestibular and egocentric attention (55), auditory coordinate location (56), and hierarchical structure processing (50). The anterior intra-parietal sulcus lobule also plays a role in manipulating objects with responsiveness to size, shape, and surfaces of specific geometries (57, 58), temporal relations with regard to grasping (57), memorizing geometry (57), coordinated defensive movements (59, 60), and writing-related functions (61). Visual-dominant neurons, found only in the anterior intra-parietal sulcus lobule, activated differently with respect to ambient light levels (60, 62). A study on patients with moderate and severe traumatic brain injuries found the anterior intra-parietal sulcus to have decreased functional connectivity in relation to sensory processing and integration and attention networks (63). Damage to the anterior intra-parietal lobule has also been shown to manifest as reduced ability to manipulate objects (62, 64) such as ideomotor apraxia (64, 65), reduced grip (64, 66), reduced tactile sensitivity (58, 67), an inability to grasp objects (57, 62, 64), difficulty visualizing object rotation (68), spatial neglect (69–71), and autotopagnosia (67). Post-concussion symptoms associated with the anterior intra-parietal sulcus lobule are somatic [e.g., light sensitivity (58, 60, 62), noise sensitivity (55, 56), motor control problems (49, 57), visual problems (62, 72)] and cognitive symptoms [e.g., feeling “slow” (63)] (Table 2).

#### 2.1.3. Broca's area

This area resides in the inferior and lateral aspect of the pars opercularis of the inferior frontal lobe, which is bordered by the Sylvian fissure and its ascending anterior ramus ventrally and the precentral sulcus dorsally in the dominant, and typically left, hemisphere (Figure 1C) (73). Broca's area is the language processing area and is fundamentally involved in the motor aspect of speech (73). Neurological signals generated from Broca's area help initiate the movement of musculature in the throat, mouth, and tongue to produce meaningful sounds and initiate complex speech (29). This area is the neural mechanism for language and plays a vital role in word decoding, language production, phonology, articulation, and ensuring proper grammar, and is associated with all language-related tasks (29, 42, 74, 75). Structurally, Broca's area can be further subdivided into two parts, Brodmann's Area (BA) 44 and BA45 (which is located in the pars triangularis of the frontal operculum of the inferior frontal lobe, which is bordered by the Sylvian fissure and its horizontal ramus ventrally and its ascending anterior ramus posteriorly), and the arcuate fasciculus pathway links Wernicke's area to Broca's area to produce the complete motor and sensory aspects of language and speech (64, 76). BA44 is more involved in language production, whereas BA45 is further involved in semantics and fluency, temporal or affective encoding (77).

Damage to Broca's area includes, but is not limited to, conduction aphasia, difficulty initiating speech, effortful speech production, difficulty forming sentences, impairment in speech melody, poor articulation, semantic and phonemic paraphasia, slurring, production of telegraphic sentences, abnormal grammatical forms, and omitting the ending of words (29, 73, 74, 76–78). The overall absence of auditory comprehension can also lead to a reduced ability to imitate other people's spoken words and difficulties with reading and writing (29, 78). One study on symptomatic adolescents at 1 month post-concussion found that Broca's Area had decreased activation during the working memory 1-back > 0-back contrast task, and symptomatic participants felt significantly more slowed down, mentally foggy, had difficulties remembering and concentrating, had a lower neurocognitive index, and complex attentional test scores (25). Post-concussion, injury of Broca's Area could manifest as cognitive symptoms [e.g., feeling “slow” or “foggy”, difficulty concentrating or remembering (25), or speech and language impairments (77, 78)] (Table 2).

#### 2.1.4. Hippocampus

The hippocampus is a symmetrically elongated brain structure that lies deep near the brain's hemispheric midline toward the splenium of the corpus callosum and follows it anteriorly but also has lateral extensions to the temporal lobe (Figure 1D). The hippocampus can be separated into head, body, and tail segments, with subdivisions of the hippocampal head that include the cornu ammonis, dentate gyrus, and the subiculum (29). The hippocampal entorhinal cortex facilitates learning, memory, emotion, and social behavior (43, 79), whereas the subiculum focuses on episodic memory functions (80). The hippocampi are a central structure that connects with and affects all brain functions (33, 79, 81, 82), with a closer influence over the premotor cortex (83), medial geniculate bodies (84), mammillary bodies (33, 79), and other deep brain structures in the diencephalon (29, 33, 85). The thalamus, hypothalamus, amygdala, mammillary body, and fornix also have key hippocampal connections to comprise the limbic system and enable memory facilitation (43, 84). Furthermore, important white matter structures such as the cingulum (33, 86), uncinate fasciculus (43, 81), and corticospinal tract (82) facilitate other functions of the hippocampus.

The main role of the hippocampi is to execute every aspect of memory (33, 42, 86), but other vital roles include learning new tasks (29), understanding verbal and spatial cues (29, 68, 87), motivation (43), egocentric and allocentric coding (88, 89), and executive function (33).

Damage to the hippocampus often manifests in a variety of memory impairments (81, 90) affecting verbal (29), spatial (29, 91), and episodic (29, 33, 79) memory functions (Table 2). Furthermore, hippocampal atrophy due to aging (92), concussions (93), and chronic stress (35) can produce cascading cell loss and/or gliosis (40) leading to myopathy, weakness, fatigue, bone decalcification, and further neural degeneration (94). Among other conditions, the sustained degradation of the hippocampus has been shown to cause amnesia (95), mild cognitive impairment (96, 97), Alzheimer's Disease (98), depression (99), and anxiety disorders (100). Since hippocampi are so crucial to learning, damage has also been shown to impair social conditioning and certain motor tasks (91). Concussions have been shown to cause cerebral blood flow and activity in the hippocampus which would be related to the presence of memory-related symptoms (13). Thus, in summary, common post-concussion symptoms related to focal hippocampus damage could be cognitive [e.g., feeling “foggy”, or difficulty remembering (13, 79)] or emotional symptoms [e.g., feeling more emotional, sadness, and nervousness (99, 100)]. Fortunately, in unilateral damage, the option of some compensation through the communication of the contralateral hippocampus, along with memory training, has been shown to initiate compensatory neuroplastic processes (33) to diminish impairments caused by pathology (80).

#### 2.1.5. Inferior parietal lobule

The inferior parietal lobule is a large symmetric lobule (i.e., grouping) on the inferior aspect of the parietal lobe below the inferior parietal sulcus (Figure 1E) (29). The inferior parietal lobule also harbors the upswing of the long, deep arcuate intra-parietal sulcus behind the lower postcentral gyrus which then slashes posteriorly across the convex surface of the parietal lobe (29). The inferior parietal lobule can be further separated into 3 subregions based on cytoarchitecture into the anterior, middle, and posterior subdivisions (101). The connectivity of the inferior parietal lobule with all major semantic areas of the brain lends itself to communicating with aspects of the temporal lobe (43, 102), occipital lobe (103), cerebellum (64), neighboring superior and anterior intraparietal lobules (33, 43, 75), sensory and motor cortices (43, 49, 102), and deep brain structures such as the insula (104), amygdala (34), medial geniculate bodies (105), and hippocampi (30). Additionally, white matter bundles such as the arcuate fasciculus (106), cingulum (33, 43), and superior longitudinal fasciculus (43, 107) form long U-shaped fibers (74) that connect Broca and Wernicke's area for the left inferior parietal lobule to aid in the language (43, 108).

Overall, the inferior parietal lobule interfaces with several areas of information convergence to facilitate a variety of sensorimotor and behavior-related actions (52, 109). Visuospatial navigation is primarily carried out by the inferior parietal lobule, which plays roles in visuomotor mechanisms (49, 110, 111), velocity/timing information (112), grasping (49), and complex tool use (49). Bilaterally, the inferior parietal lobule also plays roles in sound perception and auditory memory despite the primary auditory cortex being in Heschl's gyrus in the superior temporal lobe (103, 113), saccadic eye movements (114), egocentric decision-making (60), and emotional empathy (34). The left inferior parietal lobule is involved in speech and language processing (108) and reading and writing (75), while the right inferior parietal lobule is responsible for natural handwriting tempo (115).

Damage to the inferior parietal lobules generally causes reduced visuospatial and motor control abilities (52, 74, 86, 116), auditory agnosia (117), increased egocentric or allocentric behavior (55), and language deficits (108). Damage to the inferior parietal lobule is also related to speech pathology and shown to cause phonemic paraphasias (74), dysgraphia causing difficulties in reading and writing (52, 75, 118), disrupted phonological processing, and speech arrest (74). Specific to concussion, a study by Zuleger et al. found that the inferior parietal lobule was significantly altered post-concussion and significantly connected to the primary motor and sensory cortices and the inferior temporal gyrus; suggesting that sensorimotor, attention, cognition, and proprioception functions would be affected by concussion (119). In general, common post-concussion symptoms arising from inferior parietal lobule damage would include somatic [e.g., headaches, balance problems, dizziness, light or noise sensitivity (103, 113, 119) visual and motor problems (49, 119)] and cognitive symptoms [e.g., feeling “slow” or “foggy”, difficulty concentrating or remembering (103, 113, 119)] (Table 2).

#### 2.1.6. Insula

The insula is a large triangular region that lies deep into the lateral cerebral fissure (i.e., Fissura Sylvi), covered by the lower parietal and frontal lobes and transitions to form the temporal lobes (Figure 1F) (29, 120). The central sulcus of the insula separates the small gyri (usually 3) from the long gyri (usually 2). The insula is cytoarchitectonically distinguishable from surrounding brain regions by lamination patterns and degrees of granularity (37, 120, 121). The insula can be subdivided into three main subregions known as the anterior granular, posterior granular, and intermediate dysgranular cortices (37, 120, 121). The anterior and posterior granular cortices are a central node within the limbic, frontal, and auditory pathways (37, 121); whereas the intermediate dysgranual cortex mainly facilitates vestibular and somatic sensations (122). Apart from its subdivisions, the insula's unique anatomical position allows for communication with many brain regions including the larger frontal, parietal, temporal, and occipital lobes (120, 123), and with more distinct structures such as the amygdala (43), hippocampus (43), thalamus (124), midbrain (125), medial geniculate nucleus (126), auditory cortex (126), somatosensory cortices (37), motor cortices (127), and Broca's area (128), and also has connections to the cerebellum (124). White matter bundles connected to the insula also consist of the cingulum (43, 124), corpus callosum (129), and arcuate fasciculus (76).

Given its multiple communication pathways, the insular system carries out a variety of somatic and motor functions and is involved with emotional behaviors (120). Sensorially, the insula interprets contralateral, and occasionally ipsilateral (130), tactility (37, 43) with regard to feelings of warmth (37, 131, 132) and vibration (133). Processing information for auditory (126), taste (37, 134), and pain (37, 135, 136) stimuli are also insular somatosensory functions. Because of its connections to the motor cortex, the insula is also involved in speech production (122, 125, 134, 137), gastric motility (37, 135, 138), repetitive motor movements (43, 139), cardiovascular function (37, 140), and also to other afferent vagal nerve fibers (141). Finally, the insula may allow the production of the appropriate emotional responses to stimuli (142) and connect feelings with decision-making (120, 143). Emotions such as empathy (60), body awareness (37, 86), decision-making tasks (37, 132), and disgust (37, 144) all have insular involvement.

Pathology associated with the insula is often characterized by spontaneous somatosensory sensations that cause discomfort and pain along with a series of other impairments (Table 2). Somatosensory discomfort can include warmth and thermal sensitivity, violent and painful electric current sensations in the face, mouth, and upper limbs, abdominal heaviness, and difficulty breathing (37, 134). As the insula forms part of a central pain pathway (145), patients with insular lesions may suffer from pseudothalamic syndrome (146, 147), painful paresthesias (37), nociceptive sensitivity, analgesia/hyperalgesia (146), and difficulties processing pain (37, 148). Insular pathologies can result in auditory impairments (131), including hearing loss (105), auditory agnosia (149), or auditory hallucinations (150), olfactory (29, 42) or gustatory impairments such as unpleasant and metallic tastes (37), alterations in gastro-intestinal movement/motility and tone (130), and deficits in discriminating size, texture, and shape of objects (151). Moreover, speech-related problems can manifest as conduction aphasia (76, 103), effortful speech, articulation impairments, semantic and phonemic paraphasias, telegraphic sentences, abnormal grammatical form, and dysphonic and dysarthric speech (37, 134). Other symptoms that have been noted due to insular damage include mental confusion (130), short-term memory deficits (76), and nausea (67). Isnard et al. also found that patients with insular lesions could suffer from hypersalivation, clonic jerks in the arm or face, anxiety, compulsive swallowing, and impaired consciousness (134). Untreated insular damage could lead to empathy or emotional deficits (86), disruptive behavior disorders in adolescents (86, 152), depression in adults (34, 153), anxiety (154), and a tendency toward substance abuse (155). A recent MRI study found significantly reduced insular functional connectivity and damaged white matter connections along with slower times on completing all cognitive tasks and lower test scores compared to controls (156). Another recent study on post-concussion headaches compared persistent concussion symptoms patients with migraine patients and identified numerous functional connectivity differences between brain regions, including the insula, cingulate, temporal pole, cuneus, secondary somatosensory cortex, ventro-medial prefrontal cortex, and others (46). In summary, insular damage caused by a concussion could result in somatic [e.g., headache (46, 157), nausea, vomiting (67, 130), noise sensitivity (105, 134), and motor problems (120, 134), or bodily pain (145, 148)], cognitive (e.g., feeling “slow” or “foggy” (156), or language problems (120, 134)], or emotional symptoms [e.g., irritability or feeling more emotional (86, 142, 153)] (Table 2).

#### 2.1.7. Lateral geniculate body

The lateral geniculate bodies, also referred to as the lateral geniculate nuclei, are a pair of dense, symmetric neurons that lie directly lateral to the medial geniculate bodies (Figure 1G) (158). The lateral geniculate bodies play a significant role in relaying visual impulses from the retina by integrating pathways from the optic radiation, optic nerve, Meyer's loop, corpus callosum, brainstem, occipital cortex, and other visual-related nodes (31, 158). The lateral geniculate bodies are also considered to have a large thalamic component and thus are the first stage at which feedback signals affect visual processing (159, 160). The thalamic connection to the lateral geniculate bodies governs selective attention control related to visual inputs (158).

Pathology associated with the lateral geniculate bodies is characterized by an overall loss of visual experience, lack of visual awareness, and a reduced ability to understand visual inputs (Table 2) (158, 161). In addition, due to the link to selective attention, lateral geniculate body damage can also produce blindsight in particular areas and lead to difficulty concentrating on visual objects (158, 161, 162). A diffusion MRI case study on a 35-year-old mild traumatic brain injury female patient found auditory deficits and deafness in connection to white matter injury between the lateral geniculate body and occipital pole (163). Based on the neuroanatomical role of the lateral geniculate body and the diffusion case study, common post-concussion symptoms related to lateral geniculate body impairment could result in somatic symptoms (e.g., visual problems and sensitivity to noise and light).

#### 2.1.8. Mammillary body

The mammillary bodies are a pair of spherical structures within the inferior hypothalamus paramedian of the brain that lies directly adjacent to the rostral-anterior aspect of the brainstem (Figure 1H) (79, 164). The mammillary bodies are part of the limbic system and part of the Papez circuit, which facilitates memory and emotion (31, 79). The mammillary bodies are particularly involved in long-term memory function, word recognition, recall of episodic information, spatial processing, and the ability to understand olfactory inputs (33, 165, 166).

Damage to the mammillary bodies can lead to a variety of memory, olfactory, and spatial deficits, however, because it is considered a relay station in the Papez circuit, the bodies can undergo atrophy due to damage in its other connecting nodes such as the amygdala, fornix, hippocampus, and thalamus (31, 167) or due to chronic alcohol consumption (168). The functions of these structures have also been associated with dementia, epilepsy, schizophrenia, amnesia, and the loss of smell and/or the inability to process or understand the sense of smell (31, 165, 166). A recent article on chronic traumatic encephalopathy (CTE) found that mammillary bodies were frequently atrophied in CTE patient brains, and thus could apply to individual concussion cases (169). Post-concussion, damage to the mammillary bodies could result in somatic [e.g., headaches and loss of smell (33, 165)] and cognitive symptoms [e.g., difficulty remembering (33)] (Table 2).

#### 2.1.9. Medial geniculate body

The medial geniculate bodies, often referred to as the medial geniculate nuclei, are a pair of symmetric structures that lie directly adjacent to the brainstem and medial to the lateral geniculate nuclei (Figure 1I) (170, 171). The medial geniculate bodies can be subdivided into ventral (172), dorsal, and medial (173). Connections from the medial geniculate bodies to the inferior colliculus and the auditory cortex also form pathways to create a detailed association between speech and sound (173). The medial geniculate bodies are mainly responsible for relaying auditory impulses or sounds from the ear to the temporal lobe via acoustic radiation and the thalamus (173, 174).

Auditory frequencies processed in the medial geniculate bodies are organized such that complex and higher-order sounds are processed with the neuronally denser lemniscal pathway that integrates auditory and multisensory information, whereas secondary sounds, such as sharp responses to tones, are processed by less neuron-rich regions (171). The extralemniscal pathway then processes responses to basic tones (150, 174). The medial geniculate bodies have also been known to facilitate the efficient transmission of auditory linguistic signals in speech to preserve and perceive environmental sounds (175). Pathology associated with the medial geniculate body is generalized auditory agnosia and a reduced ability to understand auditory inputs (171, 176). Partially because of the close connection between the medial geniculate bodies and other subcortical structures such as the hippocampus and amygdala, a rodent model traumatic brain injury study found abnormally increased activity in the medial geniculate bodies in connection to auditory-induced post-traumatic stress and amygdala dysfunction (177). Based on the functions of the medial geniculate body and its connections to other subcortical structures, concussion-related damage could manifest as somatic symptoms [e.g., headaches and noise sensitivity (171, 177)] and possibly emotional symptoms [e.g., nervousness (177)] (Table 2).

#### 2.1.10. Premotor cortex

The premotor cortex spans a substantial portion of the frontal lobe and lies directly anterior to the primary motor cortex (Figure 1J) (29). The premotor cortex can be subdivided into ventral and dorsal regions and interacts with the primary motor cortex, corticospinal tract, colliculi projections, acoustic radiation, auditory cortex, basal ganglia, cerebellum, and the limbic system to generate and plan motor movements (29, 124). Information from multimodal sensory inputs is sent to the premotor cortex where spatial coordinates are transformed into an appropriate visuomotor 3D representation of space for the primary motor cortex to convert abstract goals into planned motor actions (124, 178). The premotor cortex is therefore utilized in precise, fine-motor hand movements (43, 62, 178). The premotor cortex also combines tactile, visuospatial, proprioceptive, and cognitive information to carry out specialized tasks (43, 49, 52, 60, 64, 84, 178). Studies have further shown that the premotor cortex plays roles in particular social behaviors such as language and articulation processes (52, 60), writing tasks (75), music cognition (150), early phases of learning (64), imitation and empathy (60), understanding intentions and actions (106), vigilance (179), and motivation (180).

Pathology associated with the premotor cortex mainly manifests in reduced motor control which can cause difficulties in chewing and performing facial expressions (29), performing coordinated movements (64), learning a new skilled movement (64), and ideomotor apraxia (181). Premotor cortex damage can also affect precise hand movements, errors in limb position and trajectory, and praxis in hand and finger movements (64). Due to its involvement with speech, premotor pathology can also cause hearing impairments (150), complete speech arrest (74), articulatory disturbances (182), anarthria, or dysarthria (74, 122), and in severe cases can lead to Pick's disease (64). It is also important to note that significant crosstalk occurs between the bilateral premotor cortices and the ipsilateral primary motor cortex in which symptoms can sometimes be expressed (64). A study on acutely concussed adolescents found that the premotor cortex was one of several brain regions that had significantly reduced BOLD activity during working memory tasks (183). Meanwhile, a recent study on a different group of adolescents found that increased connectivity between the default mode network (DMN) and the lateral premotor cortex was correlated with motor impairments post-concussion (184). A study on white matter projections from the corpus callosum found that concussed female athletes had a lower white matter volume and fewer tracts projecting to the premotor cortex; related symptoms were not reported but would be related to motor movements and coordination (185). Specific to post-concussion headaches, a recent resting-state fMRI study found that the premotor cortex was consistently abnormal in those with mild, moderate, and severe post-concussion headaches (157). Post-concussion symptoms that could arise from premotor cortex injury would primarily be somatic [e.g., headaches (157), nausea, balance problems, dizziness, fatigue, and motor control problems (64, 184, 185)], but could also include cognitive symptoms [e.g., feeling “slow”, language problems (60, 74, 75), or difficulty remembering (64, 183)] (Table 2).

#### 2.1.11. Primary motor cortex

The primary motor cortex is located on the superior aspect of the frontal and parietal lobes, on either side of the central sulcus and anterior to the primary somatosensory cortex, and has some anatomical overlap with the premotor cortex (Figure 1K) (29). The primary motor cortex is closely connected to the premotor cortex, somatosensory cortex, thalamus, hippocampus, corpus callosum, and brainstem to effectively perform motor movements (29, 60, 186). Closely linked by proximity and functional communication to the somatosensory homunculus (187–189), the primary motor cortex is also organized somatotopically where specific zones are responsible for directing the action of specific groups of muscles, joints, and limbs (186). The organization of the primary motor cortex begins inferolaterally with the tongue, continuing superiorly in the order of lips, squinting, and fingers, with zones for the wrist, forearm, and elbow interspersed on the superolateral aspect of the primary motor cortex, with the lower limb and foot zone located on the superomedial aspect (29, 186).

Regarding function, the primary motor cortex is responsible for the execution of voluntary bodily movement. Once a specific motor task has been decided upon, a “blueprint” for the motor task is sent to the spinal cord or the cranial nerves for task execution (29, 186). Information from the primary motor cortex is transmitted through the brainstem's pyramidal desiccations to the contralateral corticospinal tract (i.e., motor plans for the right arm are generated by the left primary motor cortex) (29, 186). To ensure proper coordination, the primary motor cortex also incorporates important sensory feedback through touch, proprioception, autonomic functions, pain, temperature, strength of muscle contractions, and audiovisual inputs (124, 189). Apart from directly controlling movements, the primary motor cortex is also involved in writing tasks (75), executive control (190, 191), imitation (60), and early phases of learning (64).

Pathology associated with the primary motor cortex traditionally follows the loss of function in contralateral muscles, muscle weakness, and reduced motor skills and muscle selectivity (192, 193). Other pathological symptoms can include impairments to gait, balance, skilled movements, muscle paresis, muscle atrophy (29, 64), facial palsy, spasticity, and hearing loss (Table 2) (150, 194). Similar to the premotor cortex, the primary motor cortex also exhibits cross-talk between hemispheres, therefore, subtle abnormalities in ipsilateral limbs may also be present (64). However, if injuries persist, limb-kinetic apraxia can develop into corticobasal degeneration and further into Pick's disease (64, 195). Fortunately, the primary motor cortex is highly adaptive and has shown a high capacity for plasticity during injury recovery (196, 197). A ^1^H-MR spectroscopy study on acutely concussed young adults found that the primary motor cortex had significantly decreased glutamate (i.e., main excitatory neurotransmitter) and *N*-acetylaspartate that correlated with symptom severity (198). Another study, using transcranial magnetic stimulation, found that concussion severity and subsequent concussions had long-term effects resulting in subclinical motor cortex dysfunction (199). The primary motor cortex is involved in many functions, and as such these post-concussion symptoms could arise as somatic [e.g., headaches, nausea, vomiting, balance problems, fatigue, and movement impairments (29, 64, 199)] or cognitive symptoms [e.g., feeling “slow” (64, 190)] (Table 2).

#### 2.1.12. Primary somatosensory cortex

The primary somatosensory cortex is a large brain region that is symmetric and directly posterior to the primary motor cortex and the central gyrus, with some anatomical overlap with the premotor cortex (Figure 1L) (29). The primary somatosensory cortex stretches from the longitudinal fissure to the Sylvian fissure (Lateral sulcus) approximately along the gyri immediately posterior to the central sulcus. The primary somatosensory cortex can be further subdivided into four cytoarchitectonic areas arranged from anterior to posterior termed Brodmann areas BA3a, BA3b, BA1, and BA2 that connect to other brain structures to process all sensory sensations of the human body (29, 200).

Mechanoreceptive somatosensory inputs from the primary sensory areas including the visual, auditory, vestibular, and other nervous systems send information through the spinal cord to the primary somatosensory cortex to contextualize sensory information to aid future motor-based decisions (29, 37). The primary somatosensory cortex is therefore processing the sensory information for proprioception (29, 55, 64, 180), vision (47, 180), motor control (201), regulating cortical excitability (67), involuntary movement activation (202), working memory (29), fast perceptual learning (203), and pain (204–206). Like the primary motor cortex, the primary somatosensory cortex is organized somatotopically in discrete zones, described by the somatosensory homunculus (187, 188). Furthermore, each subdivision has been demonstrated to have complete maps of the contralateral body surface (187). Neuroplasticity and cortical reorganization are present in the primary somatosensory cortex (207, 208), which indicates that cortical maps are in a constant state of fluctuation (203) and that neural representation is dependent on triggered stimuli (67).

Damage to the primary somatosensory cortex can cause an overall reduction in sensory input and interpretation, which can manifest as reduced tactile ability, poor grip, object manipulation, uncoordinated finger movements (29), impaired recognition of facial expressions (67, 209), and praxis errors involving orientation, limb coordination, and motor control (Table 2) (64, 201). Discomfort and pain are also commonly elicited as paresthesia, pins and needles, numbness, tingling, and warmth affecting the lips, cheek, face, tongue, upper limbs, and lower limbs (37, 134, 210). Additionally, deficits in pain processing occur where pain can be generated sporadically (94) or create phantom limb pain (67). A recent longitudinal fMRI study found that the functional connectivity of the primary somatosensory cortex significantly reduced at 1 month post-concussion but recovered after 5 months, and that improved somatosensory connectivity was correlated with symptom resolution (211). Thus, post-concussion symptoms would be expected in the presence of primary somatosensory injury as primarily somatic symptoms [e.g., headaches (46), nausea, vomiting, balance problems, light sensitivity, noise sensitivity, bodily pain, numbness, or visual problems (180, 201, 211)] (Table 2).

#### 2.1.13. Secondary somatosensory cortex

The secondary somatosensory cortex is another large brain region that is symmetric and lies directly posterior to the primary somatosensory cortex on the inferolateral aspect of the parietal lobe (Figure 1M) (29). Similar to the primary somatosensory cortex, the secondary somatosensory cortex can be further subdivided into four segments based on their cytoarchitecture and functional differences: Operculum (OP) 1 (lateral dorsal), OP2 (posterior ventral), OP3 (anterior ventral), and OP4 (anterior) (212, 213).

The secondary somatosensory cortex carries out similar sensory processing functions as the primary somatosensory cortex (29, 55, 64, 139). Also, like the primary somatosensory cortex, the secondary cortex is organized inferolaterally to superomedially in a somatotopic form in the order of face, hands, trunk, and legs (139, 213). The secondary somatosensory cortex sets itself apart in the ability to localize the origin of somatic sensations and communication with the parietal cortex (48) and limbic system (94). Moreover, the localization abilities are attributed to enlargements of representation maps (203, 208, 214) and less consistent somatotopic organization (139, 151, 215), where variability in activation improves discrimination abilities (203, 216).

Damage to the secondary somatosensory cortex often manifests in reduced psychophysical performance (203), reduced tactile sensitivity, memory problems (29), phantom limb pain (203, 217), and praxis errors (64). However, pathology is predominantly presented as diverse and unpleasant sensations of paresthesia, pins and needles, numbness, tingling, electrical current, warmth, electric discharge, and pain in the lips, cheek, face, tongue, upper and lower limbs, neck, and torso (29, 37, 62, 134). Fortunately, the secondary somatosensory cortex is known to have strong cortical reorganization abilities that drive plastic changes, along with similar contralateral somatotopy to reduce effects due to pathology (139, 151, 203). Similar to the primary somatosensory cortex, concussion-related damage to the secondary somatosensory cortex could result in somatic symptoms [e.g., headaches (46, 157), nausea, balance problems, dizziness, light sensitivity, noise sensitivity, numbness, visual problems, or bodily pain (134, 203)] (Table 2).

#### 2.1.14. Superior parietal lobule

The superior parietal lobule is a large symmetric brain region on the superior aspect of the parietal lobe and is situated directly superior to the intra-parietal sulcus and incorporates a substantial amount of the cortical parietal lobe tissue posterior to the primary motor and somatosensory cortices (Figure 1N) (218). The superior parietal lobule can be further subdivided into five subregions (218). The main function of the superior parietal lobule is to integrate multimodal somatosensory and visual inputs to create specific motor movements and is thus highly connected to motor and sensory brain regions (29, 218). In addition to motor and sensory integration, the superior parietal lobule plays a role in egocentric tasks (219, 220), emotion-relevant behavior (86, 221), and auditory association (222, 223).

Damage to the superior parietal lobule mainly manifests in visuospatial navigation impairments (68, 111) causing a variety of praxis errors (64, 75), particularly in the dark (64), such as apraxic dysgraphia (52), autotopagnosia (64, 67), poor balance (48), and poor posture (64). Superior parietal lobule pathology has also been shown to reduce attention spans in youth (86) and is strongly correlated with pathology attributed to the inferior parietal lobe (52). A study on acutely concussed adolescents performing a navigational memory task had their fMRI BOLD signal change from baseline was negatively correlated with post-concussion symptom severity in the superior and inferior parietal lobes, premotor cortex, and parahippocampus (224). Another study on post-concussion adolescents also found verbal and visual memory impairments related to the superior and inferior parietal lobules (225). Post-concussion symptoms associated with superior parietal lobe injury could include somatic [e.g., headaches (46), balance problems, dizziness, light sensitivity, motor control problems, or visual problems (48, 64, 218)] or cognitive symptoms [e.g., feeling “slow”, difficulty remembering, and confusion (224, 225)] (Table 2).

#### 2.1.15. Visual cortex

The visual cortex is a large region that covers much of the occipital lobe (Figure 1O) (29). More specifically, the primary visual cortex is located at the most posterior point of the occipital lobe, which is medial and close to the longitudinal fissure (29). The secondary and association visual areas cover most of the remaining aspects of the occipital lobe, which is superolateral to the primary visual cortex (29). Based on the description provided by Purves et al. the visual cortex can be separated into eight different brain regions, where V1 is the primary visual cortex and V2 is the secondary visual cortex, while V3, V3a, V4, ventral posterior (VP), middle temporal (MT), and middle superior temporal (MST) comprise the remaining association visual areas (226). The calcarine sulcus runs transversely through the primary visual cortex, the secondary visual cortex wraps around the primary visual cortex, V3 and V3a are superior to the secondary visual cortex, and VP and V4 are inferior to it (201). The MT and MST regions are small and slightly separated from the other visual cortex regions, found on the inferior, lateral aspects of the occipital lobe (226).

In general, the visual cortex is responsible for receiving, processing, and interpreting visual information that travels from the retina, along the optic nerve, passing through the thalamus, and arriving at the primary visual cortex (29). This includes the processing of color, brightness, shape, and motion captured with the visual sensory system (226). Visual information is also processed on the contralateral side of the brain than the eye. The cortical visual regions of V3A, MT, and MST are involved in motion perceptions (226–228), whereas V4 is involved with color interpretation and processing (Table 2) (226, 227). A study on seven acutely concussed young adults found numerous oculomotor impairments related to the visual cortex during the first-week post-injury and after 30 days (229). Another study examining concussion patients exhibiting vestibular symptoms noted vestibular, visual, and sensory processing networks to be affected (230). Due to the visual implications of concussion-related injury to the visual cortex, concussion-related visual cortex damage would primarily present as somatic symptoms [e.g., headaches, nausea, vomiting, balance problems, dizziness, light sensitivity, and visual problems (226, 229, 230)] (Table 2).

### 2.2. White matter brain regions

#### 2.2.1. Acoustic radiation

Acoustic radiation is a white matter tract that originates at the medial geniculate nucleus of the thalamus and travels anterior and lateral toward the primary auditory cortex on the transverse temporal gyri of the temporal lobe (Figure 2A) (174, 231). It is essential to transmitting auditory information from the thalamus to the temporal cortex and is therefore essential to auditory and language comprehension (174, 232, 233).

Damage to the acoustic radiation could lead to a range of auditory impairments (Table 3). Studies have shown that damage to acoustic radiation is associated with hearing and language disorders, auditory processing deficits, and decreased speech comprehension (174). More serious damage could lead to cortical (central) deafness (117, 149), environmental sound agnosia, total auditory agnosia of all sounds (149), or verbal deafness (word agnosia) (175, 176). Additionally, an individual can experience auditory hallucinations (i.e., the experience of hearing music in the absence of any external stimuli) (150) or tinnitus (234). Language impairments may be more likely if an injury occurs to the left acoustic radiation as research has shown a more substantial acoustic radiation asymmetry and predicted that the more developed left acoustic radiation may be due to language processing being performed in the left hemisphere (232). One study found acoustic radiation damage in association with concussions (235), while another found increased acoustic radiation connectivity in association with years of soccer played (236); however, neither reported associations with those abnormalities in relation to concussion symptoms. Extrapolating from those concussion-related abnormalities and the normal function of the acoustic radiation, post-concussion symptoms could include somatic [e.g., noise sensitivity (149)] or cognitive symptoms [e.g., language problems (232)] (Table 3).

**Table 3:** A summary of 10 white matter brain regions and their associated functions and concussion-related symptoms.

**Brain region**	**Associated functions**	**Concussion-related symptoms**
Acoustic radiation	• Auditory and language comprehension • Auditory processing deficits • Language processing (left hemisphere)	• Noise sensitivity • Language problems
Callosal body	• Inter-hemispheric connection • Visual • Motor • Visuospatial perception • Information processing speed and ability • Moral reasoning • Tactile and somatosensory perception • Behavior • Higher cognitive functions • Learning bimanual tasks	• Headache • Dizziness • Motor control problems • Feeling “slow” or “foggy” • Difficulty concentrating • Difficulty remembering
Cingulum	• Executive control • Attention • Episodic memory • Pain sensation • Psychiatric disorders • Depression • Anxiety • Psychosis	• Bodily pain • Nervousness • Anxiousness • Trouble falling asleep • Excessive sleep • Loss of sleep • Feeling “slow” or “foggy” • Difficulty remembering
Corticospinal tract	• Voluntary motor control	• Headaches • Balance problems • Motor control problems • Feeling “foggy” • Language problems • Irritability
Fornix	• Episodic memory • Learning capabilities • Attention	• Feeling “slow” or “foggy” • Difficulty concentrating • Difficulty remembering
Inferior occipito-frontal fascicle	• Social cognition • Episodic memory • Attention and multitasking • Behavioral-cognitive flexibility • Executive function • Decision-making • Language • Hearing • Visual conceptualization and recognition	• Visual problems • Feeling “slow” or “foggy” • Difficulty concentrating • Difficulty remembering • Language problems
Optic radiation	• Visual field • Retinal function • Light perception	• Balance problems • Light sensitivity • Visual problems • Motor control problems
Superior longitudinal fasciculus	• Dorsal ° Visuospatial attention (right) ° Motor (bilateral) • Ventral ° Attention and social cognition (right)	• Motor control problems • Feeling “slow” or “foggy” • Difficulty concentrating or remembering • Language problems
	° Language, auditory comprehension, and articulation processing (left) ° Motor (bilateral) • Posterior° Auditory and visuospatial comprehension (right)° Auditory comprehension, reading, and lexical access (left) • Arcuate fasciculus° Social and visuospatial cognition (right)° Phonological language processing (left)	
Superior occipito-frontal fascicle	• Speech • Motor • Language • Sensory • Visual field • Visuospatial cognition • Spatial working memory • Processing speed and simple reaction time	• Balance problems • Visual problems • Light sensitivity • Motor control problems • Feeling “slow” or “foggy” • Difficulty concentrating • Language problems
Uncinate fascicle	• Mood regulation • Emotional expression • Interpreting facial expressions • Learning and memory • Language (left)	• Feeling “slow” or “foggy” • Difficulty concentrating • Difficulty remembering • Confusion

#### 2.2.2. Callosal body

The callosal body, also known as the corpus callosum, is a large commissural tract that connects the left and right hemispheres by way of more than 200 million nerve fibers (Figure 2B) (237, 238). The callosal body resides in the center of the brain and connects with and crosses many other white matter tracts (31, 238). The corpus callosum can be subdivided into anterior, middle, and posterior sections, respectively, named the genu, body, and splenium of the corpus callosum (239).

Due to its substantial inter-hemispheric connection, the callosal body is essential to most facets of cognitive function. Therefore, injury to this important white matter structure could cause a wide range of cognitive and neurological complications. These could include visual, motor, and visuospatial perception, information processing speed and ability, moral reasoning, tactile and somatosensory perception, behavior, higher cognitive functions, and learning bimanual tasks (31, 238, 240, 241). White matter injury to the callosal body following a concussion has been shown extensively in research (185, 242). Due to the vast connective importance of the corpus callosum, injury to it could result in the following post-concussion symptoms, and a review of concussion-related callosal body damage suggested that somatic (e.g., headaches, dizziness, motor control problems) and cognitive symptoms (e.g., feeling “slow” or “foggy”, difficulty concentrating, or remembering) would be present, with a more serious axonal present in women (243) (Table 3).

#### 2.2.3. Cingulum

The cingulum, also referred to as the cingulum bundle, is a substantial white matter structure that nearly forms a complete circle within the medial cortex (Figure 2C) (33). From a sagittal perspective of either hemisphere, the cingulum encircles the corpus callosum with connections to the orbitofrontal regions before posteriorly traveling anterior to the body of the corpus callosum toward the occipital lobe, and then diving inferiorly and anteriorly toward the temporal pole (33, 244).

As a result of its structure, the cingulum is highly connected to various brain regions and has been linked to have important roles in executive control (81), attention (245), and episodic memory (Table 3) (33, 246–248). Additionally, the cingulum has also been linked to pain sensation processing (33, 94) and the development of psychosis or schizophrenic behavior (249), obsessive-compulsive (33, 250), anxiety (251), and depression disorders (252, 253). This is of interest specific to post-concussion assessment because anxiety and depression commonly occur following concussions (254, 255). A recent study found that concussed adolescents had significantly higher anxiety which was also associated with lower neurite density index in their bilateral cingulum and bilateral forceps minor, with older female adolescents with the cingulum differences (256). Another study found that adults with persistent concussion symptoms had significantly reduced fractional anisotropy of their cingulum which also significantly correlated with the number of symptoms and the self-paced saccades task outcome (257). Additionally, one study found that concussed adolescents who were experiencing lower sleep quality had lower white matter neurite density index across 18 of the 19 tracts examined, with significant findings present in the cingulum, optic radiation, and the superior longitudinal fasciculus (258). Finally, a study found a complex set of cingulum bundle abnormalities in concussed adolescents that was correlated with compromised memory and learning scores (259). Thus, post-concussion symptoms related to cingulum injury could be somatic [e.g., bodily pain (94)], cognitive [e.g., Feeling “slow” and difficulty remembering (259)], emotional [e.g., nervousness and anxiousness (256, 257)], or sleep symptoms [e.g., trouble falling asleep and sleeping more or less than usual (258)] (Table 3).

#### 2.2.4. Corticospinal tract

The corticospinal tracts are well-documented bilateral white matter structures that descend from the motor cortex, travel through the medullary pyramid in the brainstem, and then cross to continue descending contralaterally down the spinal cord to the dorsolateral funiculus (Figure 2D) (260). Thus, the left and right corticospinal tracts travel contralaterally within the spinal cord.

The corticospinal tracts are essential to motor control including spinal reflexes and motor neuron control (261). Thus, one of the primary deficits associated with impairment of this region is reduced voluntary motor control (262, 263). With a concussion, injury to the corticospinal tracts could affect motor control from the neck down. Injury to the corticospinal tracts, within the brain or spinal cord, could also lead to ipsilaterally impaired proprioception, paralysis, decreased muscle tone, spasticity, power production, and mass (264, 265). A study on white matter integrity in retired professional American-style football players found significantly increased axial diffusivity in the superior longitudinal fasciculus, corticospinal tract, and anterior thalamic radiations; however, the neuropsychological function was only compared to the superior longitudinal fasciculus (266). Although not directly compared, the retired athletes reported worse memory, executive function, language, sensory, behavior, constitutional, and headache scores (266). Post-concussion, injury of the corticospinal tract could manifest primarily as somatic symptoms[e.g., headaches, balance problems, and motor control problems (261, 262, 266)], but could also present as cognitive [e.g., feeling “foggy” and language problems (266)] or emotional symptoms [e.g., irritability (266)] (Table 3).

#### 2.2.5. Fornix

The fornix is a thin, arched white matter structure within the medial aspect of the cerebral hemispheres (Figure 2E) (79, 267). Due to the arched structure, the fornix can be separated into several sections including the alveus, subiculum, fimbria, crura, body, and columns (267). The fornix is a major hippocampal output tract and resultantly travels from the medial temporal lobe regions, where the alveus is formed medially to the inferior aspect of the temporal horn of the lateral ventricle (267). The alveus bundles together to form the fimbria, which curves posteriorly and superiorly, before forming the crura which curves anteriorly and superiorly (79, 267). The forneal crura travel underneath the splenium of the corpus callosum and project to connect to form the structure known as the dorsal hippocampal commissure (79, 267). The crura come together and form the forneal body, which arches superior to the thalamus and travels anteriorly before splitting, at the anterior commissure, into the left and right columns that descend into the anterior forebrain (79, 267).

As the primary white matter structure connected to the hippocampus, the fornix is closely related to memory and learning capabilities. Thus, damage to the fornix could involve decreased episodic memory function, learning capabilities, and attention impairment (Table 3) (79, 267). Several concussion and mild traumatic brain injury studies have found decreased fornix microstructural integrity and volume following an injury that was correlated with injury severity (268–270). Furthermore, atrophy of the fornix has been linked to several neurodegenerative diseases such as Alzheimer's Disease, Parkinson's Disease, Multiple Sclerosis, epilepsy, and schizophrenia (79). A study on adults with persistent concussion symptoms found the fornix to have significantly reduced fractional anisotropy compared to healthy controls and was significantly correlated with lower processing speed and reaction time (271). Another study on adult women^∧^ with persistent concussion symptoms found that reduced FA values were correlated with higher total Graded Symptom Scale Checklist scores (272). Finally, a study by de Souza et al. found that decreased fractional anisotropy and increased mean diffusivity were correlated with worse executive function and immediate, visual, and verbal memory performances (273). Based on the healthy function of the fornix and the results of those concussion studies, a concussion-related fornix injury could present as cognitive symptoms [e.g., feeling “slow” or “foggy”, difficulty concentrating, or remembering (271, 273)] (Table 3).

#### 2.2.6. Inferior occipito-frontal fascicle

The inferior occipito-frontal fascicle is one of the long and highly connected white matter bundles in the human brain, however, its distinction from other white matter structures has been a point of controversy for decades (Figure 2F) (274, 275). Fortunately, the evolution of diffusion magnetic resonance imaging (dMRI) has recently allowed for highly detailed fiber tracking of the inferior occipito-frontal fascicle that can be corroborated with cadaveric brain dissections (123, 275, 276). The posterior aspect of the inferior occipito-frontal fascicle originates in the lateral, inferior portion of the occipital lobe and travels through the occipital lobe lateral to the ventricle horns (123, 276). The tract remains lateral through the temporal lobe before veering medially into the anterior portion of the insular short gyri and terminating anteriorly in the orbitofrontal cortex (123, 276). This white matter tract is also close in proximity and functional involvement to the inferior longitudinal fasciculus.

Based on its anterior-to-posterior anatomical structure, the inferior occipito-frontal fascicle is associated with many important functions that can involve anatomically distant regions (123). Furthermore, dMRI studies have found the inferior occipito-frontal fascicle to be specifically involved in various tasks. Due to its anterior connections within the frontal lobe, Brodmann's Area (BA) 10, the inferior occipito-frontal fascicle is associated with many complex cognitive functions such as social cognition, episodic memory, attention, and multitasking (123, 191). Obsessive-compulsive disorder, and its associated behavioral-cognitive flexibility, executive function, and decision-making deficits, has been linked to the inferior occipito-frontal fascicle's connection of the frontal lobe with the temporal and occipital lobes (123, 277, 278). Additionally, the fronto-temporal fiber section of the inferior occipito-frontal fascicle is associated with language and hearing, where the left region connects to Broca–Wernicke language centers (279) and is affected by auditory verbal hallucinations in individuals with schizophrenia (280). Finally, there has been some evidence of the inferior occipito-frontal fascicle being implicated with visual conceptualization and recognition (124). To the best of our knowledge, no study has directly explored post-concussion symptoms in specific relation to the inferior occipito-frontal fascicle. Therefore, potential post-concussion symptoms are extrapolated from the normal function of this white matter tract. Thus, injury to the inferior occipito-frontal fascicle could present as somatic (e.g., visual problems) or various cognitive symptoms [e.g., feeling “slow” or “foggy”, difficulty concentrating or remembering (123, 191), or language problems (279)] (Table 3).

#### 2.2.7. Optic radiation

Optic radiation is a vital white matter tract responsible for transmitting visual information from the eye to the visual cortex in the occipital lobe (Figure 2G) (281). Optic radiation is a hook-shaped white matter structure that originates at the lateral geniculate nucleus, a transfer point in the thalamus receiving visual information from the optic tracts and terminates in the primary visual cortices in the occipital lobe (281).

The visual information transmitted to the primary visual cortex via optic radiation is contralateral to the eyes. Thus, the main deficit associated with optic radiation damage is visual impairment (282). An injury to the optic radiation can lead to decreased visual field and light perception (283, 284) and reduced retinal function (285). A case study on two mild traumatic brain injury patients with complex visual field loss had significantly abnormal volume and diffusion characteristics (286). Another study found that adults with persistent symptoms after a mild traumatic brain injury experienced significantly reduced fractional anisotropy of the optic radiation that correlated with light sensitivity (287). Finally, a study on visuomotor function post-concussion found that significantly reduced optic radiation fractional anisotropy correlated with poorer visuomotor performance (288). Based on the normal role of the optic radiation and the previously mentioned concussion studies, a concussion could injury the optic radiation and present with somatic symptoms [e.g., balance problems, motor control problems (288), sensitivity to light, or visual problems (286, 287)] (Table 3).

#### 2.2.8. Superior longitudinal fasciculus

The superior longitudinal fasciculus is another large white matter structure that due to its many branches and tracts has left researchers and clinicians with some ambiguity surrounding its exact anatomy (Figure 2H) (289). Generally, the superior longitudinal fasciculus connects most cortical regions of the parietal lobe to the frontal lobe, with some temporal connections as well (289). Due to the numerous names associated with the tracts and segments of the superior longitudinal fasciculus, one recent study (289) proposed a simplified naming convention separating the superior longitudinal fasciculus into four segments named dorsal, ventral, posterior, and arcuate fasciculus segments (289–292). The dorsal segment would be what has been previously referred to as the superior longitudinal II, the ventral segment to the arcuate fasciculus anterior and superior longitudinal fasciculus III, the posterior segment to the arcuate fasciculus posterior and temporoparietal segment of the superior longitudinal fasciculus, and the arcuate fasciculus to the arcuate fasciculus or the arcuate fasciculus long segment (289). The dorsal segment originates in the inferior parietal lobe and terminates in the superior and middle frontal gyri, while the ventral segment also originates in the inferior parietal lobe, slightly anterior and inferior to the dorsal segment, and terminates in the middle and inferior frontal gyri (289). The posterior segment originates in the superior, middle, and inferior temporal gyri and terminates within the inferior and superior parietal areas (289). Finally, the arcuate fasciculus segment originates across the superior, middle, and inferior temporal gyri before traveling posteriorly and arcing around the Sylvian fissure and insula to terminate anteriorly in the posterior aspects of the inferior and middle frontal gyri (289). To note also, the inferior longitudinal fasciculus follows a similar path through the brain as the superior longitudinal fasciculus and is often examined in the context of concussions.

Based on the four-segment naming convention proposed by Nakajima et al., each segment can be related to specific cognitive functions related to the cortical regions it connects (Table 3) (289). As proposed by Nakajima et al. and based on previous literature, the function of each superior longitudinal fasciculus segment can be classified as bilaterally or hemisphere-specific (289). The dorsal segment is involved in visuospatial attention in the right hemisphere and bilaterally in motor control, the ventral segment is involved in attention and social cognition in the right hemisphere, language, auditory comprehension, and articulation processing in the left hemisphere, and motor control bilaterally, the posterior segment is involved in auditory and visuospatial comprehension in the right hemisphere and auditory comprehension, reading and lexical access in the left hemisphere, and the arcuate fasciculus is involved in social cognition and visuospatial cognition in the right hemisphere and phonological language processing in the left hemisphere (289). A recent study on varying degrees of traumatic brain injuries found that the fractional anisotropy of superior longitudinal fasciculus was positively correlated with executive function, memory, and attention (293). Therefore, in summary, common post-concussion symptoms related to superior longitudinal fasciculus would include some somatic symptoms [e.g., motor control (289)], but primarily cognitive symptoms [e.g., feeling “slow” or “foggy”, difficulty concentrating or remembering, or language problems (289, 293)] (Table 3).

#### 2.2.9. Superior occipito-frontal fascicle

The superior occipito-frontal fascicle is a long association white matter tract that connects the frontal and occipital cortices (Figure 2I). The tract travels parallel to the corticospinal tracts and corpus callosum between the corticospinal tracts and the lateral ventricles, and inferiorly to the corpus callosum (294, 295). Anterior and posterior to the corpus callosum, the superior occipito-frontal fascicle projects superiorly (295).

Due to the location, length, and connection of the frontal and occipital cortices, the superior occipito-frontal fascicle is associated with several functions. A study of 90 awake glioma craniotomy patients found that the superior occipito-frontal fascicle had mapping points associated with specific characteristics for speech disorder (27.2%), motor disorder (24.7%), language disorder (16.1%), sensory disorder (15%), and several other functions with less distinction (295). The study also found that the superior occipito-frontal fascicle was positively associated with the visual field, visuospatial cognition, and spatial working memory (295). Another study found that young adults with Multiple Sclerosis had reduced processing speed and simple reaction time correlated with negative abnormalities in their superior occipito-frontal fascicle, corpus callosum, and corticospinal tracts (296). Thus, confirming that the superior occipital-frontal fascicle relays important information among the visual, motor, and executive functioning brain regions (296). Similar to the inferior occipito-frontal fascicle, there has been little to no research to this point on the direct effects of concussion on the superior occipito-frontal fascicle. Therefore, based on the healthy role of this brain structure and symptom presentation found in relation to abnormalities from other conditions, the damage could present as somatic [e.g., balance problems, visual problems, sensitivity to light, motor control problems (295)] or cognitive symptoms [e.g., feeling “slow” or “foggy”, difficulty concentrating, or language problems (296)] (Table 3).

#### 2.2.10. Uncinate fascicle

The uncinate fascicle is an important white matter tract that connects the temporal cortex with the prefrontal cortex (Figure 2J). The structure originates in the temporal pole and travels posteriorly to the amygdala before the body of the uncinate fascicle curves superiorly through the external capsule medial to the insular cortex, and then has a unique hook shape to turn antero-medially toward the prefrontal cortex (297–299). The body of the uncinate fascicle then branches in three directions toward the lateral orbital gyri, frontopolar cortex, and subgenual cingulate cortex (297, 298).

Based on the anatomical location of this structure and its close connection to the prefrontal cortex and amygdala, it has been shown that the uncinate fascicle is involved in mood regulation, emotional expression, and depression (252, 297), and even problems interpreting facial expressions (300). The uncinate fascicle also passes close to the hippocampus and due to its presence in the temporal lobe has been associated with learning and memory (301, 302). Furthermore, it has also been linked to language due to its position within the parietal lobe (82, 301, 303). One study found that the uncinate fascicle had significantly decreased fractional anisotropy post-mild traumatic brain injury correlated with worse memory test performances (304). Other studies have found uncinate fascicle damage that correlated with attention, memory, cognitive reaction time, and learning in concussion or mild traumatic brain injury patients (305–307). Therefore, a concussion-related injury to the uncinate fascicle would likely be exhibited as cognitive symptoms [e.g., feeling “slow” or “foggy”, difficulty concentrating or remembering, and confusion (304–307)] (Table 3).

### 2.3. Cerebellum

The cerebellum is a unique part of the brain that is located posteriorly within the skull, inferior to the occipital lobe. Despite the cerebellum being substantially smaller than the cerebrum, it has been shown to contain about four times as many cells as the entire cerebrum (308). Similar to the cerebrum, the cerebellum is separated into two hemispheres by the cerebellar vermis and consists of three lobes; the anterior, posterior, and flocculonodular lobes (26, 308, 309). Each lobe is also separated into lobules, which can be further separated into folia (308). The cerebellum has 12 lobules known as I, II, III, IV, V, VI, Crus I, Crus II, VIIb, VIII, IX, and X on each cerebellar hemisphere (Figure 3) (26, 309). These lobule regions are organized with distinctions between lobules from the superior to the inferior external surface (26, 309). There are also an additional set of cerebellar regions that are located along the proximal and medial aspect of the cerebellum between the two lobes, known as the cerebellar vermis (26, 309), but they will not be discussed in this review as they are smaller and have had little concussion-related research examining them to date.

From a cognitive and functional perspective, our understanding of the cerebellum has undergone a revolution. For around 200 years, the cerebellum was believed to be strictly involved with motor control (3). However, the advent of functional medical imaging techniques has allowed for the realization that the cerebellum is involved in motor control, language, attention, working memory, emotion, and social processing (3, 310, 311). Functional MRI studies have shown that the cerebellum and its lobules can be subdivided into two motor regions and three non-motor regions (267). Based on a summary article by Guell and Schmahmann, lobules I–VI make up the first motor region, lobule VI and crus I make up the first non-motor region, crus II and VIIb make up the second non-motor region, lobule VIII makes up the second motor region, and lobules XI and X make up the third non-motor region (310). Although the primary functions of the cerebellum, motor, attentional/executive, and default mode network activation, are expressed quite generally across the lobules, the less involved functions of emotional, vestibular, language, and social processing are exhibited in more specific cerebellar regions (Table 4) (310). Emotional processing has been found close to the cerebellar vermis and thus is more associated with medial aspects of the lobules VI, crus I, and crus II (310, 312). Vestibular activation has been found in the verbal aspects of lobules crus I, crus II, and VII, and lobules IX and X (313); however, this activation may overlap with visual, emotional, and other motor functions (310). Similar to the lateralization of the cerebrum, language activation is lateralized contralaterally to the cerebrum and found in the right cerebellar hemisphere (310, 314). Finally, social cognition overlaps greatly with the default mode network activation in the cerebellum, which can be generally seen in lobules crus I, crus II, XI, and X (310, 312).

**Table 4:** Cerebellar regions and their associated functions and concussion-related symptoms are based primarily on the summary provided by Guell and Schmahmann (310).

**Brain region**	**Associated functions**	**Concussion-related symptoms**
I	• Motor	• Balance problems • Feeling “slow” • Motor control problems
II	• Motor	• Balance problems • Feeling “slow” • Motor control problems
III	• Motor	• Balance problems • Feeling “slow” • Motor control problems
IV	• Motor	• Balance problems • Feeling “slow” • Motor control problems
V	• Motor	• Balance problems • Feeling “slow” • Motor control problems
VI	• Motor • Attention • Executive functions • Working memory • Attentional and executive processing	• Balance problems • Feeling “slow” • Motor control problems • Difficulty remembering • Difficulty concentrating
Crus I	• Attention • Executive functions • Default mode processing • Emotion • Vestibular • Social cognition	• Balance problems • Dizziness • Irritability • Sadness • Nervousness • More emotional • Feeling “slow” or “foggy” • Difficulty concentrating
Crus II	• Attention • Executive functions • Default mode processing • Emotion • Vestibular • Social cognition	• Balance problems • Dizziness • Irritability • Sadness • Nervousness • More emotional • Feeling “slow” or “foggy” • Difficulty concentrating
VII	• Attention • Executive functions • Default mode processing • Vestibular	• Balance problems • Dizziness • Feeling “slow” or “foggy” • Difficulty concentrating
VIII	• Motor	• Balance problems • Feeling “slow” • Motor control problems
IX	• Attention • Executive functions • Default mode processing • Vestibular • Social cognition	• Balance problems • Dizziness • Feeling “slow” or “foggy” • Difficulty concentrating
X	• Attention • Executive functions • Default mode processing • Vestibular • Social cognition	• Balance problems • Dizziness • Feeling “slow” or “foggy” • Difficulty concentrating

Concussions have been linked to cerebellar abnormalities. Functional connectivity of the cerebellum is significantly associated with the number of previous concussions some have had and concussion recovery time (315). A recent study on symptomatic and asymptomatic acutely concussed youth found that cerebellar inflammation was associated with acute symptom severity and that the cerebellum had significantly increased functional connectivity with the precuneus and inferior parietal lobule which was not present in asymptomatic concussed participants (316). Meanwhile, an older study by Jantzen et al. found that cerebellar abnormalities in concussed college football players were associated with worse movement sequencing, working memory, and motor control performance (317). However, due to the vast number of functions associated with the cerebellum, further research is required to determine the risk of injury to the cerebellum during a concussive event. However, common post-concussion symptoms could be somatic [e.g., balance problems, fatigue, bodily pain, motor control problems (186, 317)], cognitive [e.g., feeling “slow”, difficulty concentrating (310, 317)], or emotional in nature [e.g., irritability, feeling more emotional (310, 314)] (Table 4).

## 3. Discussion

This review aimed to highlight the intimate connection between post-concussion symptoms in the event of concussion-related damage to specific brain regions. Complete incorporation of all brain structures and post-concussion symptoms was not feasible, especially considering spatial resolution limitations of medical imaging techniques for very small brain regions, and thus this review is understandably not exhaustive at that scale. Further research is also required to connect common post-concussion symptoms more concretely to specific brain regions.

### 3.1. Clinical concussion diagnosis

The diagnosis of a concussion is primarily based on emergency physician examination, patients reporting their symptoms, and potentially basic medical imaging, but could include further testing if more serious brain damage is suspected (5). Despite efforts to improve concussion recognition and management protocols (4, 318), diagnosis can still be highly variable due to the subjectivity of patient self-reporting, varying concussion assessment guidelines, and clinician interpretation (319). Some of the tests used to diagnose concussions can be self-administered, while some must be administered by a trained clinician (320, 321). Commonly used concussion diagnosis tests include Immediate Post-Concussion Assessment and Cognition Tool (ImPACT) (322), Sport-related Concussion Assessment Tool-−5th Edition (SCAT5) (323), CogSport (324), the Post-Concussion Symptom Scale (28), and the Rivermead Post-Concussion Symptoms Questionnaire (325), with others described in detailed review articles (326).

### 3.2. Potential to incorporate research into clinical practice

Although symptom-based diagnosis and recovery tracking remains the clinical gold standard, structural computed tomography (CT) and magnetic resonance imaging (MRI) scans are used to rule out skull fractures and bleeding in more serious concussion and traumatic brain injury cases (327). However, advances in MRI have provided powerful insights into brain function following a concussion using safe, non-invasive, objective, and reproducible methods. Routine clinical 3-dimensional T1-weighted and T2-weighted MRI scan protocols usually fail to identify any concussion-related damage (327). Susceptibility-weighted imaging (SWI) might depict sheering and microbleed injuries.

The MRI scan techniques used in research with promising clinical potential include diffusion MRI (dMRI), functional MRI (fMRI), MR spectroscopy (MRS), and arterial spin labeling (ASL). Those techniques have all been shown to identify microstructural, functional, metabolic, or tissue perfusion alterations, respectively, in acute and chronic concussion patients (27, 327). Instead of MRI being limited to ruling out serious brain bleeding and skull fractures, symptom-based testing can be supplemented by highly sensitive MRI techniques. These more advanced MRI techniques can provide extensive information but are not yet implemented for clinical concussion diagnoses. Incorporation of these techniques can inform clinicians of a concussion patient's current brain health, track recovery, and objectify post-concussion symptoms (i.e., nausea) with data acquired from highly reproducible MRI scans and post-scan analyses.

## 4. Conclusion

The heterogeneity of concussions has challenged clinicians and patients alike. However, connecting the functional and anatomical brain characteristics may highlight opportunities for personalized treatments and be useful in determining an individual concussion patient's recovery prognosis. Unfortunately, identifying that a concussion patient is suffering from light sensitivity and has visual cortex and optic radiation abnormalities, for example, currently does not guarantee their complete recovery. However, the summed knowledge we present here aims to allow clinicians and researchers alike to focus their efforts on developing injury and patient-specific treatment options.

## Author contributions

ED, NS, DK, and MN were involved in the conceptualization of this work. ED and NS were responsible for the investigation and collection of information, as well as the writing of the original draft. ED, NS, CD, DK, SU, and MN were involved in the editing and review of the manuscript. ED was responsible for project administration. MN was responsible for supervision. ED and MN were responsible for the visualization of the published article. All authors contributed to the article and approved the submitted version.
